# Impact of Comorbidities and Skin Diseases on Post-Vaccination Reactions: A Study on COVID-19 Vaccinations in Poland

**DOI:** 10.3390/jcm13206173

**Published:** 2024-10-17

**Authors:** Izabela Jęśkowiak-Kossakowska, Paulina Nowotarska, Patrycja Grosman-Dziewiszek, Adam Szeląg, Benita Wiatrak

**Affiliations:** 1 Department of Pharmacology, Faculty of Medicine, Wroclaw Medical University, Mikulicza-Radeckiego 2, 50-345 Wroclaw, Poland; patrycja.grosman-dziewiszek@umw.edu.pl (P.G.-D.); adam.szelag@umw.edu.pl (A.S.); 2 Department of Biostructure and Animal Physiology, Wroclaw University of Environmental and Life Sciences, Norwida 25/27, 50-375 Wroclaw, Poland; paulina.nowotarska@upwr.edu.pl

**Keywords:** COVID-19 vaccination, adverse events, comorbidities, skin diseases, vaccine safety, Poland

## Abstract

**Background:** The COVID-19 pandemic necessitated rapid and widespread vaccination efforts, which proved critical in reducing the severity and mortality of the virus. However, the interplay between vaccinations, pre-existing skin conditions, and other comorbidities still needs to be explored. This study investigated the occurrence and severity of adverse events following immunization (AEFIs) with COVID-19 vaccines in individuals with chronic skin diseases and comorbidities within a Central European cohort. **Methods:** An anonymous online survey was conducted between May 2022 and February 2023, targeting students and employees of universities in Wrocław, Poland. A total of 513 respondents were analyzed, focusing on AEFIs following the first, second, and third doses of COVID-19 vaccines and the effects of COVID-19 on conditions such as atopic dermatitis, psoriasis, vitiligo, acne vulgaris, rosacea, and various comorbidities. **Results:** COVID-19 vaccination effectively protected against severe disease across all doses. The analysis revealed no significant impact of either COVID-19 infection or vaccination on the course of selected skin diseases and comorbidities. The reporting of AEFIs to the Sanitary Inspection was notably low. The Moderna and Pfizer mRNA-based vaccines were associated with a higher reported number of AEFIs, particularly after the second and third doses, compared to AstraZeneca, which exhibited fewer adverse events after subsequent doses. **Conclusions:** COVID-19 vaccination is both safe and effective, even in patients with pre-existing skin conditions and comorbidities. Vaccine selection may benefit from considering individual health profiles, and better reporting of AEFIs is needed to enhance vaccine safety monitoring.

## 1. Introduction

Coronavirus disease (COVID-19) is a highly infectious viral disease caused by the severe acute respiratory syndrome virus (SARS-CoV-2) [1]. The rapidly increasing number of deaths around the world has created an urgent need to develop an effective vaccine against COVID-19 [2].

The WHO declared COVID-19 a pandemic on 11 March 2020 [1] and the first case of the disease was recorded in Poland on 4 March 2020. Universal vaccination against COVID-19 commenced in 2021. According to data from the Ministry of Health in Poland, from the beginning of the epidemic to the end of 2021, 4,119,652 people were ill with COVID-19 (10.8% of the Polish population), of which 25,680 became sick again. In 2021 alone, 2,813,781 people (7.4% of the Polish population) became ill. In 2020 and 2021 the COVID-19 epidemic was the main cause of the increased number of deaths in Poland. In 2021, 92.8 thousand people died of COVID-19, which accounted for nearly 17.8% of all deaths. This was an increase of almost 9.2 percent in the percentage of total deaths caused by COVID-19, compared to 2020 (in 2020, 41,451 people died of COVID-19, which accounted for nearly 8.7% of all deaths during this period). In 2021 the COVID-19 epidemic affected men and city dwellers to a greater extent. The reason for this phenomenon may be, among others, that men in Poland neglected preventive examinations. A total of 43,119 women (46.5%) and 49,661 men (53.5%) died of COVID-19 in 2021. The victims of the COVID-19 epidemic were predominantly older individuals, with the majority of people over 65 years of age (approximately 82.5%). In the age group of 80 years or more, more women than men died from COVID-19. In the remaining age groups, the majority of the dead were men. It is worth noting that, in 2020, people under the age of 24 years accounted for a very small percentage of those who died due to SARS-CoV-2 coronavirus infection. (according to Central Statistical Office data, source https://stat.gov.pl/obszary-tematyczne/zdrowie/zdrowie/zdrowie-i-ochrona-zdrowia-w-2021-roku,1,12.html accessed on 8 July 2024).

COVID-19 infections can range from asymptomatic infections to severe, acute respiratory failure and death [3]. Vaccination is a key method of fighting the pandemic [4]. Due to the severity of the disease, numerous vaccines were quickly developed and introduced to prevent coronavirus infections [5]. The vaccines targeted the SARS-CoV-2 spike protein and have been administered to almost a billion patients worldwide [6]. While numerous vaccines have been developed globally, such as Sinopharm (China) and Sputnik V (Russia), this study focused on the vaccines that were primarily available in Poland during the study period: Pfizer-BioNTech, Moderna, AstraZeneca, and Johnson & Johnson. The Pfizer BioNTech and Moderna vaccines were most frequently used in Poland [7]. The first batch of vaccines against COVID-19 arrived in Poland on 26 December 2020. The preparations were distributed to 72 nodal hospitals throughout the country, where vaccinations were carried out for people most at risk of infection. The first person was vaccinated on December 27 at the hospital of the Ministry of Internal Affairs and Administration in Warsaw. By the end of 2021, 21.1 million people across the country (55.7% of the population) had been fully vaccinated (according to Central Statistical Office data, source: https://stat.gov.pl/obszary-tematyczne/zdrowie/zdrowie/zdrowie-i-ochrona-zdrowia-w-2021-roku,1,12.html, accessed on 8 July 2024).

Vaccinations helped reduce the occurrence of infections throughout the population [8]. Monitoring for side effects is an essential part of investigating the safety of each vaccine by providing the public with accurate information on the potential side effects that can be expected in the weeks following vaccination, and can also minimize the impact of unexpected symptoms associated with future vaccine acceptance [9,10].

An adverse event following immunization (AEFI) is any adverse event occurring after vaccination, and most AEFIs are products of the immune response stimulated by vaccines [11]. AEFI is a medical condition that occurs within four weeks of receiving the vaccine. Current reports on AEFIs as of 15 June 2023, indicate that since the beginning of vaccination (27 December 2020), 18,781 adverse post-vaccination reactions have been reported to the State Sanitary Inspectorate, of which 15,625 were mild—such as redness and short-term pain at the injection site (https://www.gov.pl/web/szczepimysie/niepozadane-odczyny-poszczepienne, accessed on 8 July 2024).

COVID-19 infections are dangerous, especially for the elderly and patients with comorbidities [12]. In this study, the effectiveness of the first, second, and third doses of the COVID-19 vaccine in protection against COVID-19 was assessed.

Also, it determined the occurrence and severity of post-vaccination reactions after vaccination against COVID-19. In addition, this study evaluated the impact of COVID-19 disease and COVID-19 vaccinations on the course of selected skin diseases such as atopic dermatitis, psoriasis, vitiligo, acne vulgaris and, rosacea, as well as selected comorbidities such as heart disease, Hashimoto, rheumatoid arthritis, diabetes, atherosclerosis, Crohn’s disease, ulcerative colitis, and cancer. This study represents one of the few comprehensive analyses conducted in a Central Europe context, providing unique insights into the interaction between COVID-19 vaccination and chronic skin diseases and comorbidities. The findings are crucial for guiding public health strategies and clinical practices, particularly in populations with pre-existing conditions, an area that has been largely under-researched until now. To date, few scientific studies have been published on the effects of COVID-19 and COVID-19 vaccinations on these conditions, with most being case reports or review articles. Another aim of this study was to verify the actual incidence of post-vaccination reactions during vaccination against COVID-19, as well as to assess whether AEFIs after vaccination against COVID-19 are still underreported.

## 2. Materials and Methods

### 2.1. Study Design, Population, and Sampling

An anonymous study was conducted in the form of an electronic survey, which began in May 2022 and ended in February 2023. The Bioethics Committee of the Medical University of Wrocław (KB357/2022) approved this study. The survey consisted of 37 questions. A link to the electronic survey was sent to employees and students of the Medical University of Wrocław and the communication departments of the Wrocław University of Environmental and Life Sciences, Wrocław University of Science and Technology, the University of Wrocław and the University of Economics in Wrocław, the District Pharmaceutical Chambers, and the District Medical Chambers; a link to the electronic survey was also posted on various online forums interested in health issues. A total of 513 responders completed the survey.

### 2.2. Measures

The inclusion criterion for the study was the age of the respondent over 19 years. The exclusion criterion was if the group of vaccinated people qualified for a vaccination with a vaccine other than Pfizer, AstraZeneca, Moderna, or Johnson & Johnson. This study also characterized (1) the social characteristics of the studied population, such as gender (female, male), education (vocational, student, PhD student, secondary education, and higher education), belonging to a specific age group (19–30, 31–40, 41–50, 51–60, and over 60 years of age); (2) occurrence of skin disease; (3) presence of comorbid diseases; (4) smoking; (5) regarding COVID-19, such as being test-confirmed and unconfirmed with COVID-19, determined on the basis of symptoms and circumstances, and the course of COVID-19 (asymptomatic, mild—mild upper respiratory tract complaints, elevated body temperature not exceeding 38 °C, cough or shortness of breath, treatment at home, without contacting a doctor, moderate-fever > 38 °C, persistent dry cough, dyspnea, pulmonary involvement visible on imaging tests, outpatient treatment in contact with a doctor, severe-hospital treatment, and very severe-hospital treatment with the use of a ventilator); (6) severity of skin diseases after contracting COVID-19; (7) severity of comorbid diseases after contracting COVID-19; (8) increased hair loss after contracting COVID-19 (up to approx. 3 to 4 months after infection); (9) post-treatment reactions to vaccination such as symptoms occurring after the first, second, and third dose of the vaccine, comparison of the severity of symptoms after the first, second, and third dose of the vaccine; (10) course of COVID-19 after the third dose of the vaccine; (11) severity of skin diseases after COVID-19 vaccination; (12) severity of comorbid disease after COVID-19 vaccination; (13) reporting the post-vaccination reactions to the Sanitary Inspection; (14) during a long period after vaccination against COVID-19 (at least 3 months), did circulatory system disorders occur?; (15) at least 3 months after vaccination against COVID-19, did renal dysfunction occur?; (16) over the long term after vaccination against COVID-19 (at least 3 months), did uncontrolled weight gain occur?

### 2.3. Statistical Analysis

Statistical analyses were performed with Statistica v13.0. Pearson’s chi-square tests were used to compare the differences between the different subgroups.

## 3. Results

### 3.1. Study Sample Characteristics

**Figure 1:**
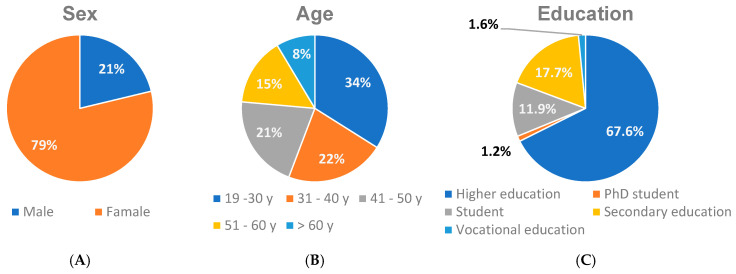
The characteristics of the study group: (**A**) sex, (**B**) age, and (**C**) education.

The contents of the questionnaire included social characteristics, such as education, and gender age groups; a graphic summary is presented in Figure 1. The overwhelming majority of women took part in the survey (404). Moreover, the majority of respondents who took part in the survey had a higher education (347). The age groups of respondents were evenly distributed: 19–30 years (174), 31–40 years (112), 41–50 years (106), 51–60 years (77), and over 60 years old (44). A graphic summary is presented in Figure 1. Most respondents did not smoke cigarettes (445).

### 3.2. Incident of COVID-19

COVID-19 confirmed via test occurred in 183 (35.7%) respondents and 127 (24.8%) respondents suffered from COVID-19 not confirmed via a test. However, 212 (39.3%) respondents marked the answer that they did not know whether they had COVID-19 which was not confirmed via a test.

When asked about the course of COVID-19, the answers were asymptomatic (29, 5.7%), mild (237, 46.2%), moderate (36, 7.0%), severe (2, 0.4%), very severe (1, 0.2%), and 208 (40.5%) responders did not suffer from COVID-19. Based on the answers provided to this questionnaire, a total of 305 (59.5%) respondents were ill with COVID-19. This did not coincide with the answers given to the previous questions, where the total number of people suffering from COVID-19 was determined to be 310 people (60.4% of respondents), because 183 people had confirmed COVID-19 test results, and 127 people had the presence of COVID-19 confirmed based on symptoms and circumstances but no test was performed. We assumed that the most reliable number of people who suffered from COVID-19 to be 305 people because the course of COVID-19 had to be determined, which made the answer to this question more reliable.

#### 3.2.1. Severity of Selected Skin Disease Following COVID-19 Infection

A total of 143 respondents (27.8%) reported having selected skin diseases, including atopic dermatitis, psoriasis, acne vulgaris, rosacea, vitiligo, and alopecia areata (Table 1). Additionally, some respondents had combinations of these conditions.

Among the 47 respondents with atopic dermatitis, 32 had contracted COVID-19. Notably, 10 of these individuals reported a worsening of their dermatitis following infection. Six respondents indicated a deterioration in their condition based solely on self-observation, while four required medical consultation, with one respondent necessitating a change in pharmacotherapy due to exacerbation of the disease. Interestingly, six of the respondents who experienced a worsening of atopic dermatitis were also afflicted with another skin disease, such as acne vulgaris (4), psoriasis (1), or rosacea (1).

Psoriasis severity increased in four out of thirteen respondents with the condition following COVID-19 infection. Three of these cases were identified through self-observation, while one required medical consultation and subsequent pharmacotherapy adjustment.

Of the 54 respondents with acne vulgaris, 38 had contracted COVID-19. Among these, 35 reported no change in their condition, while three noted a worsening of symptoms. This exacerbation was confirmed through self-observation in two cases and by a physician in one.

For respondents with rosacea, six out of the sixteen had contracted COVID-19, with only one experiencing a worsening of symptoms, which was confirmed by a physician. The remaining five reported no change.

Alopecia areata was reported by two respondents, with only one contracting COVID-19. This respondent noted no worsening of symptoms. Similarly, none of the six respondents with vitiligo reported any exacerbation following COVID-19 infection.

#### 3.2.2. Severity of Selected Comorbid Disease after Contracting COVID-19

In the study group, 74 respondents reported having comorbidities, including Hashimoto’s disease, heart disease/hypertension, rheumatoid arthritis, diabetes, cancer, atherosclerosis, multiple sclerosis, or ulcerative colitis (Table 2), as well as combinations of these conditions such as Hashimoto’s disease and heart disease/hypertension (4), Hashimoto’s disease and diabetes (3), diabetes and heart disease/hypertension (2), diabetes and atherosclerosis (1), rheumatoid arthritis and heart disease/hypertension (1), rheumatoid arthritis and Hashimoto’s disease (1), atherosclerosis and heart disease/hypertension (1), cancer and heart disease/arterial hypertension (1), and more complex combinations like diabetes, atherosclerosis, and heart disease/arterial hypertension (1), or digestive disorder, diabetes, heart disease/arterial hypertension, atherosclerosis, and cancer (1).

A total of 49 respondents were diagnosed with Hashimoto’s disease (Hashimoto’s disease only (41), Hashimoto’s disease and heart disease/hypertension (4), Hashimoto’s disease and diabetes (3), and Hashimoto’s disease and rheumatoid arthritis (1)). Among these, 29 contracted COVID-19, with three respondents reporting a worsening of their Hashimoto’s (two of whom had this confirmed following medical consultation, and one required a change in pharmacotherapy as a result).

In turn, 50 respondents were suffering from heart disease/hypertension (heart disease/hypertension only (39), heart disease/hypertension and Hashimoto’s (4), heart disease/hypertension and diabetes (2), heart disease/hypertension and atherosclerosis (1), heart disease/hypertension and cancer (1), heart disease/hypertension and rheumatoid arthritis (1), heart disease/hypertension, diabetes, and atherosclerosis (1), and heart disease/hypertension, diabetes, atherosclerosis, cancer, and Crohn’s (1)). Out of these, 29 contracted COVID-19, with five respondents experiencing a worsening of their heart disease/hypertension (three required a change in pharmacotherapy following medical consultation, while two based this assessment on self-observation).

Out of seven respondents with rheumatoid arthritis, three contracted COVID-19, with two respondents reporting a worsening of their condition based on self-observations. Among the six respondents with cancer, three contracted COVID-19, but their disease did not worsen.

#### 3.2.3. Increased Hair Loss after Contracting COVID-19 (Up to Approx. 3 to 4 Months after Infection)

A total of 76 respondents reported experiencing increased hair loss following COVID-19 infection, while 294 respondents did not notice any such increase. However, in this question, only 144 respondents answered that they had not suffered from COVID-19. This contrasts with earlier responses, where 208 respondents had reported not suffering from COVID-19.

### 3.3. COVID-19 Vaccine Preparations and Adverse Reaction after Vaccination

#### 3.3.1. COVID-19 Vaccine Preparations

According to respondents’ answers, 87.3% (448) of them were vaccinated against COVID-19, while 12.7% (65) were not vaccinated. Across all age groups, the most commonly chosen vaccine was Pfizer, accounting for 85.0% (381) of the vaccinated respondents (*p* = 0.4). Less frequently chosen vaccines included AstraZeneca (7.8%, 35 respondents), Moderna (5.8%, 26 respondents), and Johnson & Johnson (1.1%, five respondents).

#### 3.3.2. Adverse Reaction after Vaccination

Post-vaccination reactions were categorized based on their occurrence after the first, second, and third doses of the vaccine. The severity of symptoms was compared across these three doses, and an assessment was made to determine whether COVID-19 infections occurred after vaccination.

##### Post-Vaccination Reactions after the First Dose of the COVID-19 Vaccine and COVID-19 Disease after the First Dose of the COVID-19 Vaccine

Among respondents who received the first dose of the vaccine, the most common side effect, reported by 57.6% (258) of respondents, was an intense reaction at the injection site, including swelling, redness, and pain. Less frequently reported side effects included fatigue (39.1%, 175 respondents), malaise (35.9%, 161), elevated body temperature (36.6–38 °C) (26.8%, 120), and headache (24.6%, 110). Other side effects following the first dose included pain and enlargement of regional lymph nodes (10.5%, 47), fever above 38 °C (8.3%, 37), convulsions (6.0%, 27), nausea (4.0%, 18), fainting (1.8%, eight), diarrhea (1.3%, six), cough (1.3%, six), vomiting (1.1%, five), migraine (1.1%, five), and allergic reactions such as hives, tearing, runny nose, generalized rash, erythema multiforme, or asthma attack (0.7%, three). Additionally, 20.4% (90) of respondents did not experience any side effects after receiving the first dose of the vaccine.

Increased hair loss, occurring within approximately 3 to 4 months after the first dose of the COVID-19 vaccine, was reported by 33 vaccinated individuals. Conversely, 415 respondents did not experience increased hair loss.

Regarding COVID-19 infection following the first dose of the vaccine, 381 respondents (85.0%) did not contract the virus. However, among those who did contract the virus after the first dose, 19 respondents (28.4%) experienced asymptomatic COVID-19, 41 (61.2%) had mild symptoms, six (9.0%) had moderate symptoms, and one respondent experienced very severe symptoms.

##### Post-Vaccination Reactions after the Second Dose of the COVID-19 Vaccine and COVID-19 Disease after the Second Dose of the COVID-19 Vaccine

A total of 442 respondents received the second dose of the COVID-19 vaccine. Among them, 28.3% (125) reported no post-vaccination reactions. The most common side effect, reported by 50.5% (223) of respondents, was an intense reaction at the injection site, including swelling, redness, and pain. Other frequently reported side effects included fatigue (35.3%, 156 respondents), malaise (34.2%, 151), headache (23.8%, 105), and increased body temperature of 36.6 to 38 °C (23.5%, 104). Additional side effects following the second dose included enlargement and soreness of regional lymph nodes (10.0%, 44), fever above 38 °C (9.5%, 42), convulsions (6.6%, 29), nausea (3.8%, 17), migraine (2.7%, 12), fainting (1.1%, five), cough (0.8%, four), diarrhea (0.9%, four), vomiting (0.5%, two), abscess at the injection site (0.5%, two), skin ecchymosis (0.2%, one), bruising of the limbs (0.2%, one), anaphylactic shock (0.2%, one), and allergic reactions, such as hives, tearing, runny nose, generalized rash, erythema multiforme, or asthma attack (0.2%, one). Only six respondents did not receive the second dose of the COVID-19 vaccine.

Increased hair loss, occurring within approximately 3 to 4 months after receiving the second dose of the vaccine, was reported by 26 respondents (5.9%). In contrast, 413 respondents (94.1%) did not observe an increased hair loss after the second dose. Several respondents among those vaccinated with the second dose did not answer this question.

Regarding COVID-19 infection after the second dose, 347 respondents (78.5%) did not contract the virus. However, among those who did contract the virus, 24 respondents (25.3%) experienced asymptomatic COVID-19, 60 (63.2%) had mild symptoms, and 11 respondents (11.6%) had moderate symptoms.

##### Post-Vaccination Reactions after the Third Dose of the COVID-19 Vaccine and Incidence of COVID-19 after the Third Dose of the COVID-19 Vaccine

A total of 367 respondents received the third dose of the COVID-19 vaccine. Among them, 42.2% (155) reported no post-vaccination reactions. The most common side effect was a severe reaction at the injection site, including swelling, redness, and pain, reported by 38.9% (143) of vaccinated respondents. Other less frequently reported side effects included malaise (28.0%, 103 respondents), fatigue (26.6%, 98), headache (16.0%, 59), increased body temperature of 36.6 to 38 °C (15.8%, 58), and enlargement and pain of regional lymph nodes (8.4%, 31). Additional side effects included fever above 38 °C (6.0%, 22), convulsions (3.0%, 11), nausea (2.2%, eight), migraine (1.1%, four), fainting (0.5%, two), cough (0.5%, two), diarrhea (0.3%, one), and allergic reactions such as hives, tearing, runny nose, generalized rash, erythema multiforme, and asthma attacks (0.3%, one). Notably, 80 respondents (17.9%) among those vaccinated with the first dose did not receive the third dose of the COVID-19 vaccine.

Increased hair loss within approximately 3 to 4 months after receiving the third dose was reported by 25 respondents (6.8%). Regardless of age, there was no significant increase in hair loss after the third dose of the vaccine (*p* = 0.009, n = 373). In contrast, 341 respondents (93.2%) did not observe increased hair loss after the third dose.

Regarding COVID-19 incidence after the third dose, 266 respondents (72.7%) did not contract the virus. However, among those who did contract the virus, 17 respondents (5.9%) experienced asymptomatic COVID-19, 74 (25.8%) had mild symptoms, and nine respondents (3.1%) had moderate symptoms.

##### Comparison of the Severity of Post-Vaccination Symptoms after Administration of Different COVID-19 Vaccines

**Figure 2:**
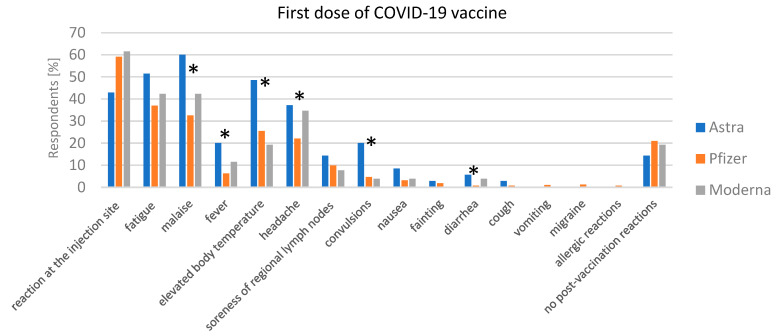
First dose of COVID-19 vaccine adverse reactions. The percentage of reported adverse reactions after the first dose of AstraZeneca, Pfizer, and Moderna vaccines. Statistically significant differences (*p* < 0.05) are marked with “*”.

The analysis of adverse reactions following the administration of COVID-19 vaccines from AstraZeneca, Pfizer, and Moderna revealed key trends and statistically significant findings. After the first dose (Figure 2), the most common reactions for AstraZeneca were malaise (60%), fatigue (51.43%), and an elevated body temperature (48.57%). Fever was reported by 20% of respondents, and injection site reactions by 42.86%. A notable 14.29% of individuals reported no adverse reactions after the first dose. For Pfizer, the most frequent reactions were injection site reactions (59.06%), fatigue (37.01%), and malaise (32.54%), with fever occurring in 6.3% of cases. Additionally, 21% of respondents reported no adverse reactions. Moderna showed similar trends, with injection site reactions (61.54%), fatigue (42.31%), and malaise (42.31%) being the most common. Fever was reported in 11.54% of cases, and 19.23% of individuals experienced no adverse reactions.

**Figure 3:**
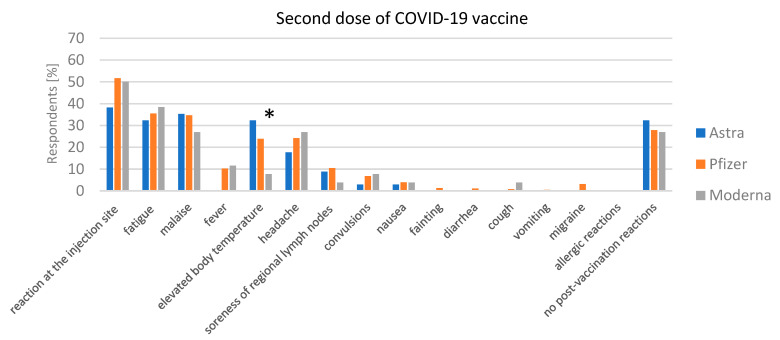
Second dose of COVID-19 vaccine adverse reactions. Comparison of the frequency of adverse reactions after the second dose of AstraZeneca, Pfizer, and Moderna vaccines. Statistically significant differences (*p* < 0.05) are marked with “*”.

After the second dose (Figure 3), there was a clear reduction in adverse reactions for all three vaccines. For AstraZeneca, malaise was reported by 35.29%, fatigue by 32.35%, and elevated body temperature by 32.35%, with no cases of fever reported. Importantly, 32.35% of individuals experienced no adverse reactions after the second dose, a statistically significant increase compared to the first dose. In the case of the Pfizer vaccine, injection site reactions were reported by 51.71%, fatigue by 35.43%, and malaise by 34.65%. Fever was present in 10.24% of cases and 27.82% of respondents reported no adverse reactions. Moderna also showed a reduction in adverse effects, with injection site reactions reported by 50%, fatigue by 38.46%, and malaise by 26.92%. Fever was reported by 11.54%, and 26.92% of individuals reported no adverse reactions after the second dose.

**Figure 4:**
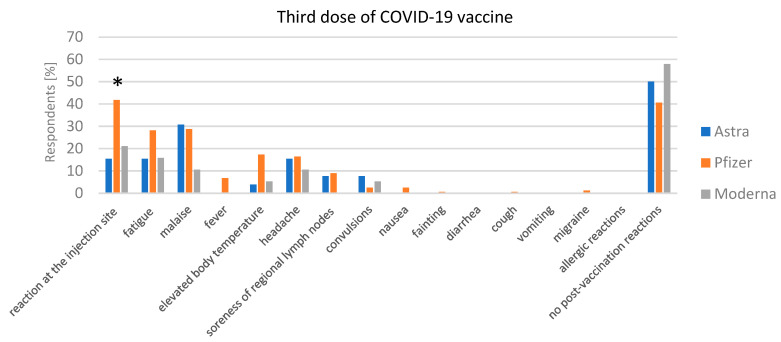
Third dose of COVID-19 vaccine adverse reactions. Reported adverse reactions after the third dose of AstraZeneca, Pfizer, and Moderna vaccines. Statistically significant differences (*p* < 0.05) are marked with “*”.

By the third dose (Figure 4), adverse reactions further decreased. For AstraZeneca, malaise was reported by 30.77%, while fatigue and injection site reactions were each reported by 15.38%. Notably, 50% of individuals reported no adverse reactions, a statistically significant increase. In the case of the Pfizer vaccine, injection site reactions were reported by 41.8%, fatigue by 28.17%, and malaise by 28.79%, while fever was present in 6.81% of cases. A significant 40.56% of individuals reported no adverse reactions after the third dose. Moderna showed the most pronounced reduction in adverse reactions, with injection site reactions reported by 21.05%, fatigue by 15.79%, and malaise by 10.53%. No cases of fever were reported after the third dose, and 57.89% of individuals experienced no adverse reactions, a statistically significant increase compared to earlier doses.

Across all three vaccines, the data demonstrate a consistent decrease in the frequency of adverse reactions with subsequent doses, and an increasing percentage of individuals reporting no adverse reactions, particularly after the third dose. These trends, especially the reduction in malaise, fatigue, and fever, as well as the significant increase in the percentage of people reporting no adverse effects, were found to be statistically significant in many instances (*p* < 0.0001), underscoring the overall improvement in tolerance after repeated vaccination.

##### Comparison of the Severity of Post-Vaccination Symptoms after Receiving the First and Second Dose of the COVID-19 Vaccine

There was no difference in the severity of post-vaccination symptoms after receiving the second dose of the vaccine for 164 respondents (37.1%). Additionally, 157 respondents (35.5%) reported experiencing milder post-vaccination symptoms after the second dose compared to the first dose. Conversely, 121 respondents (27.4%) experienced more severe symptoms after the second dose than after the first.

When asked which symptoms were more severe after the second dose compared to the first, respondents identified the following: fatigue (81), malaise (75), severe reaction at the injection site such as swelling, redness, and pain (69), increased body temperature of 36.6 to 38 °C (48), headache (47), fever (35), pain and enlargement of regional lymph nodes (24), convulsions (15), nausea (8), migraine (6), diarrhea (3), fainting (2), cough (2), vomiting (2), allergic reaction (2), and an injection site abscess (1). A total of 236 respondents (53.4%) did not experience any intensification of post-vaccination symptoms after the second dose.

##### Comparison of the Severity of Post-Vaccination Symptoms after Receiving the Second and Third Dose of the COVID-19 Vaccine

There was no difference in the severity of post-vaccination symptoms after receiving the third dose of the vaccine for 139 respondents (40.0%). Additionally, 160 respondents (43.7%) reported milder symptoms after the third dose compared to the second dose. Conversely, 67 respondents (18.3%) experienced more severe symptoms after the third dose than after the second.

When asked which symptoms were more severe after the third dose compared to the second, respondents identified the following: malaise (50), fatigue (47), severe reaction at the injection site (36), headache (31), increased body temperature (22), soreness and enlargement of regional lymph nodes (21), fever (15), convulsions (6), nausea (2), cough (2), fainting (1), migraine (1), and allergic reaction (1). A total of 249 respondents (67.8%) did not experience any intensification of symptoms after the third dose.

##### Comparison of the Severity of Post-Vaccination Symptoms after Receiving the Subsequent Doses of the COVID-19 Vaccine

Differences between post-vaccination reactions reported by respondents after the first, second, and third doses were also analyzed, with a division into individual vaccines. The results of the survey on adverse effects following COVID-19 vaccinations, including the AstraZeneca, Pfizer, and Moderna vaccines, revealed statistically significant differences in reported adverse reactions between doses. Patients vaccinated with the Johnson & Johnson vaccine were excluded from the analysis due to the small sample size of this group. A statistically significant (*p* < 0.0001) decrease in the number of reported adverse effects was observed after each subsequent dose, while the percentage of individuals reporting no reactions increased, which correlates with the observed decline in reactions.

**Figure 5:**
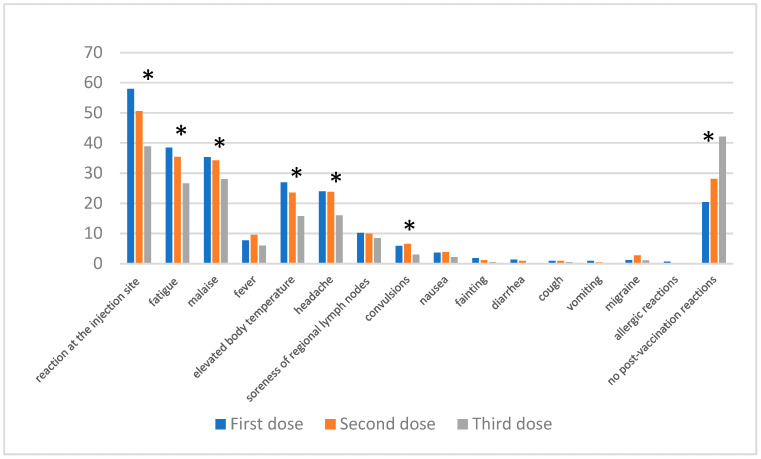
Post-vaccination reactions were reported after the 1st, 2nd, and 3rd doses of vaccine in all vaccinated respondents. “*” denotes statistical significance between the reported adverse events of individual doses.

For all vaccines combined (Figure 5), the most commonly reported adverse effects after the first dose were injection site reactions (57.92%), fatigue (38.46%), and malaise (35.29%); these differences were statistically significant. After the second dose, there was a decrease in these symptoms; fatigue was reported by 35.36% (*p* = 0.001) of individuals, and malaise by 34.24% (*p* = 0.05). After the third dose, these numbers continued to decline, with fatigue reported by 26.63% and malaise by 27.99%. A statistically significant (*p* < 0.0001) decrease was also observed in cases of elevated body temperature of 7.69% after the first dose, 9.52% after the second dose, and 5.98% after the third dose. Simultaneously, an increasing number of individuals reported no adverse effects: 20.36% after the first dose, 28.12% after the second, and as many as 42.12% after the third dose, which represents a statistically significant difference (*p* < 0.0001).

**Figure 6:**
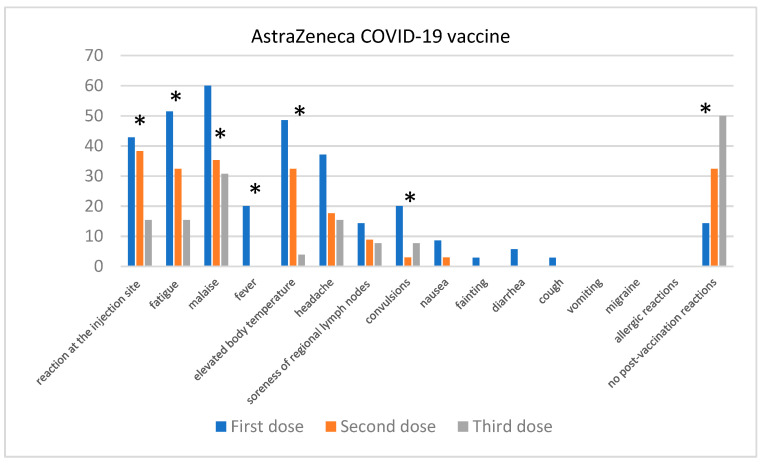
Post-vaccination reactions reported after 1st, 2nd, and 3rd dose of AstraZeneca vaccine. “*” denotes statistical significance between the reported adverse event in the individual doses.

After the first dose of the AstraZeneca vaccine (Figure 6), the most commonly reported symptoms were malaise (60%), fatigue (51.43%), headaches (48.57%), fever (20%), and elevated body temperature (48.57%). A statistically significant decrease in these symptoms was observed after the second dose; fatigue (*p* = 0.013) was reported by 32.35% of individuals, malaise (*p* = 0.03) by 35.29%, with no cases of fever (*p* = 0.0015), and elevated body temperature (*p* < 0.0001) reported by 32.35%. After the third dose, these symptoms were even rarer with fatigue (15.38%), malaise (30.77%), no cases of fever, and elevated body temperature reported by 3.85% of individuals. Convulsions (*p* = 0.05) were reported by 14.29% of respondents after the first dose, but were not recorded after the second or third doses. At the same time, there was a statistically significant increase in the number of individuals reporting no adverse effects (*p* = 0.01): 14.29% after the first dose, 32.35% after the second, and 50% after the third (*p* < 0.0001).

**Figure 7:**
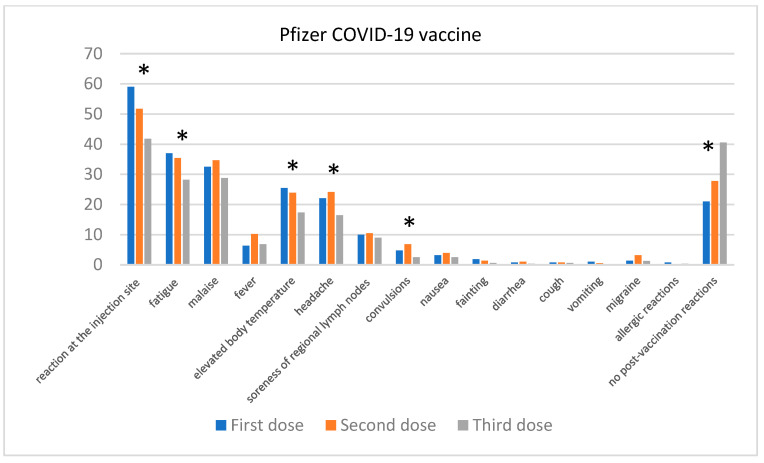
Post-vaccination reactions were reported after the 1st, 2nd, and 3rd doses of the Pfizer vaccine. “*” denotes statistical significance between the reported adverse events of individual doses.

Similar trends were observed with the Pfizer vaccine (Figure 7). After the first dose, the most commonly reported symptoms were injection site reactions (59.06%), fatigue (37.01%), and malaise (32.54%). A statistically significant decrease in reported symptoms was observed after the second dose; fatigue (*p* = 0.033) was reported by 35.43% of individuals and malaise (*p* = NS) by 34.65%. After the third dose, the further decline in reported adverse effects was even more pronounced; fatigue was reported by 28.17% of individuals and malaise by 28.79%. The number of cases of elevated body temperature (*p* = 0.026) also increased from 6.3% after the first dose to 6.81% after the third dose, which was also statistically significant (*p* = 0.0085). Convulsions (*p* = 0.026) were reported by 1.05% of individuals after the first dose but were not recorded after the third dose. The percentage of individuals reporting no adverse effects increased from 21% after the first dose to 40.56% after the third, representing a statistically significant difference (*p* < 0.0001). A statistically significant difference (*p* = 0.037) was also observed in the frequency of reported headache: 22.1% after the first dose, 24.2% after the second dose, and 16.4% after the third dose.

**Figure 8:**
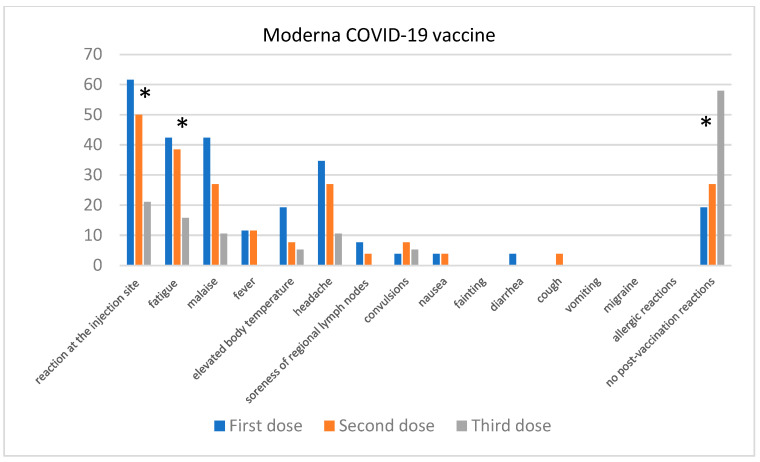
Post-vaccination reactions were reported after the 1st, 2nd, and 3rd doses of the Moderna vaccine. “*” denotes statistical significance between the reported adverse events of individual doses.

In the case of the Moderna vaccine (Figure 8), the most commonly reported symptoms after the first dose were injection site reactions (61.54%), fatigue (42.31%), and malaise (42.31%). After the second dose, a statistically significant decrease in reported symptoms was observed; fatigue (*p* = NS) was reported by 38.46% of individuals, and injection site reactions (*p* = 0.024) by 50%, and malaise (*p* = 0.05) by 26.92%. After the third dose, the number of reported adverse effects was even lower: fatigue (15.79%), injection site reactions (21.05%), and malaise 10.53%. It is also important to note the significant decrease in cases of elevated body temperature (*p* = NS) from 11.54% after the first dose to 0% after the third. Simultaneously, after the third dose, 57.89% of individuals reported no adverse effects, which was also statistically significant (*p* < 0.0001).

In summary, the results of the analysis indicate a statistically significant reduction in the frequency of reported adverse effects after each subsequent dose of COVID-19 vaccines. The percentage of individuals reporting no adverse effects increased significantly with each dose, correlating with the observed decline in reported reactions

##### Reporting an Undesirable Post-Vaccination Reaction to the Sanitary Inspection

A total of 448 respondents received the first dose of the COVID-19 vaccine. Only 91 respondents reported no post-vaccination reactions, meaning that 357 respondents experienced side effects. It would be expected that these individuals would report their reactions to the Sanitary Inspection. However, only six respondents reported their post-vaccination reactions, representing just 1.7% of the total reactions.

##### The Impact of Vaccination against COVID-19 on Selected Skin Diseases

The number of vaccinated respondents and the number of cases in which the skin disease worsened are presented in Table 3.

Out of 47 responders suffering from atopic dermatitis, 41 responders received the COVID-19 vaccine. In 36 responders, vaccination against COVID-19 did not affect the course of atopic dermatitis. In eight responders, atopic dermatitis worsened after COVID-19 vaccination, which was confirmed in five responders based on their own observations and in three patients based on medical consultation including, in one case, the need to change pharmacotherapy.

Thirteen responders suffering from psoriasis were vaccinated against COVID-19 (three responders were not vaccinated). In three responders, psoriasis worsened after vaccination against COVID-19, but in two responders the severity was confirmed based on their own observations, and in one responder based on a doctor’s consultation with the need to change pharmacotherapy.

Out of 54 responders suffering from acne vulgaris, 47 responders were vaccinated against COVID-19, and eight responders were not vaccinated. Among 47 responders who suffer from acne vulgaris and were vaccinated against COVID-19, five responders experienced an intensification of acne vulgaris after vaccination, four responders based on their own observations, and one responder based on a doctor’s consultation. Among the 16 respondents suffering from rosacea, 14 were vaccinated, and none of them experienced a worsening of their condition following vaccination.

Both respondents suffering from alopecia areata were vaccinated against COVID-19, and neither experienced a worsening of their condition after COVID-19 vaccination. Out of eight respondents with vitiligo, six were vaccinated against COVID-19. In one case, the condition worsened, confirmed via medical consultation, which required a change in pharmacotherapy.

##### The Impact of Vaccination against COVID-19 on Selected Comorbidities

The number of cases in which exacerbation of comorbidities occurred after vaccination is presented in Table 4. It is noteworthy that individuals with comorbidities were more likely to receive COVID-19 vaccinations compared to those without underlying health conditions (93.8% vs. 85.5%, *p* = 0.02).

Out of 49 respondents suffering from Hashimoto’s disease, 45 were vaccinated against COVID-19. In four respondents, the course of Hashimoto’s disease worsened; three cases were confirmed via a doctor’s consultation, which necessitated a change in pharmacotherapy, and one case was based on the respondent’s own observation.

Among the 50 respondents with heart disease or hypertension, 48 were vaccinated against COVID-19. In seven respondents, the condition worsened; three cases were confirmed via medical consultation, requiring a change in pharmacotherapy, and four cases were based on personal observation.

All seven respondents suffering from rheumatoid arthritis were vaccinated against COVID-19. In two respondents, the condition worsened, as noted based on personal observation.

Out of six respondents with cancer, five were vaccinated against COVID-19. Only one respondent experienced a worsening of their cancer, requiring a change in pharmacotherapy. However, this individual also suffered from other comorbidities including diabetes, atherosclerosis, heart disease/hypertension, and a digestive disorder.

##### Occurrence of Circulatory System Disorders, Kidney Dysfunction, and Uncontrolled Weight Gain in the Long Term after Vaccination against COVID-19 (At Least 3 Months)

In the long term following of COVID-19 vaccination patients (at least 3 months), most respondents did not experience any circulatory system problems (363). However, some respondents observed issues such as increased fatigue during normal activities (55), heart palpitations (32), episodic increases in heart rate (20), elevated blood pressure (11), including two cases requiring a change in pharmacotherapy, hypertension (9), continuously increased heart rate (7), and elevated blood pressure requiring a change in pharmacotherapy (2).

Similarly, in the long term after COVID-19 vaccination, the majority of respondents did not report any kidney function issues (402). A minority of respondents reported symptoms long after vaccination, including increased thirst (28), back pain in the kidney area (19), reduced urine output (6), and lack of thirst (3).

Moreover, the majority of respondents (388) did not experience uncontrolled weight gain in the long term following COVID-19 vaccination (at least 3 months), with only 60 respondents reporting such an effect.

## 4. Discussion

The conducted survey confirmed that in most cases, AEFI is still not reported to the State Sanitary Inspection, as was also shown in a previously conducted study [13]. Consequently, the safety data sheets of COVID-19 vaccines cannot be adequately updated with newly occurring side effects. Moreover, this study showed that regardless of age, administration of the first, second, or third dose of the COVID-19 vaccine ensures high effectiveness in protection against COVID-19. Meta-analysis indicated that in older adults, COVID-19 vaccines were effective in preventing severe acute respiratory syndrome [14]. The two-dose regimen is clearly superior to the single-dose regimen in terms of immunogenicity and efficacy [15]. The survey showed no significant impact of COVID-19 disease and COVID-19 vaccination on selected skin diseases and comorbidities. This study broke new ground by systematically analyzing a large and diverse population, revealing that, contrary to initial concerns, COVID-19 vaccination does not exacerbate chronic skin conditions or comorbidities. This contradicts earlier case reports and highlights the importance of large-scale studies in understanding the true impact of vaccination.

A detailed analysis of adverse effects based on the vaccine manufacturer was conducted. Significant differences in the frequency of AEFI, depending on the vaccine type were identified, with an overall trend showing a reduction in adverse events with subsequent doses, especially in the case of the Moderna vaccine. mRNA vaccines, such as Moderna and Pfizer, represent a new generation of vaccine technology, which may explain the slower adaptation of the body, particularly after the initial dose. In contrast, AstraZeneca, based on a viral vector platform, showed relatively fewer reported adverse effects after subsequent doses, suggesting a faster immune system adaptation to this technology. This provides a clearer understanding of how different vaccine mechanisms impact post-vaccination reactions.

The stratification of adverse effects by vaccine type offers valuable insights for healthcare professionals and patients when choosing vaccines based on individual health conditions and risk factors. This is particularly important for populations with comorbidities or those more prone to certain adverse events.

However, while this analysis provides a comparison of adverse effects across different vaccines, further research is needed, especially regarding the smaller group who received the Johnson & Johnson vaccine. Additionally, future studies should explore whether the composition of different vaccines plays a role in exacerbating or mitigating adverse reactions in individuals with chronic health conditions.

In people with comorbidities and people aged 60 years and older in the Chinese population, the incidence of adverse events after COVID-19 vaccination was similar to that in the general population [16]. Another study in the Chinese population confirmed that the COVID-19 vaccine is safe for patients aged ≥ 60 years with hypertension and/or diabetes [17]. In the German population, especially people over 80 years of age, there was exceptionally good tolerance and equally good effectiveness in vaccination against COVID-19. The gender comparison showed that women were more likely to experience side effects after vaccination [18]. In the Mexican population, an association was found between older age and a higher incidence of comorbidities, the occurrence of AEFI after vaccination and COVID-19 infection [19].

In an Italian cross-sectional study, it was shown that the likelihood of experiencing an adverse reaction after vaccination was lower with age and after the second dose of the vaccine. AEFI was more frequently observed among women and people who had COVID-19 at least once [20]. While women are significantly more likely to experience AEFI than men, the course and burden differ only slightly between the sexes [21]. Similar results from women undergoing vaccination against COVID-19 were presented in a study in the Spanish population [22].

In the German population, it was also identified that young age, male gender, and comorbid factors such as dementia, depression, anxiety disorders, hypertension, and obesity are associated with an increased occurrence of post-vaccination adverse events diagnosed in medical clinics [23]. Hypertension, central obesity, and smoking are associated with lower antibody (Ab) titers after COVID-19 vaccination. Perhaps people with hypertension, central obesity, and who are smokers may benefit from earlier vaccine boosters [24]. A meta-analysis conducted by a team from India showed a higher mortality rate in patients with COVID-19 in the presence of hypertension (39%) and asthma, therefore it was concluded that comorbidities should accelerate regular vaccination. However, mRNA-based COVID-19 vaccines and potentially AstraZeneca may cause an increased risk of myocarditis in younger people, but the absolute incidence remains low [25,26].

In a French population of elderly cancer patients who were vaccinated, the incidence of COVID-19 was much rarer than in the unvaccinated population [27]. Moreover, a study conducted in the United States compared cancer patients with healthy people in response to vaccination against COVID-19. The differences were small in the number of reported adverse events and according to the studies conducted, active cancer treatment had little impact on the side effects [28].

All individuals suffering from inflammatory bowel disease are recommended to be vaccinated against COVID-19 due to the increased risk of severe acute respiratory syndrome, including those undergoing immunosuppressive therapy [29]. The incidence of local symptoms after the first, second, and third doses of the vaccine was comparable between patients with ulcerative colitis and Crohn’s disease in the Japanese population [30]. Studies in the Czech population have shown the safety of the vaccination against COVID-19 in patients with inflammatory bowel disease treated with immunosuppressive and/or biological drug therapy [31]. Other data also provide experimental evidence indicating the effectiveness and safety of repeated vaccinations against COVID-19 in patients with rheumatic diseases [32,33,34,35].

In the case of skin diseases, vaccinated patients with atopic dermatitis showed a significantly reduced risk of infection and mortality related to COVID-19. Exposure to immunosuppressive drugs did not impair protection against SARS-CoV-2 infections following vaccination [36]. The conducted study did not demonstrate the impact of the disease and vaccination against COVID-19 on the severity of atopic dermatitis, although the severity of atopic dermatitis after contracting COVID-19 occurred in 10 people, of which four had the severity of atopic dermatitis confirmed in consultation and in one person it was necessary to change the pharmacotherapy for atopic dermatitis. In six people, atopic dermatitis worsened after vaccination against COVID-19, which was confirmed in five people based on their own observations, and in only one person based on a doctor’s consultation. A study by a Korean team showed that female gender and dermatological diseases, including allergic contact dermatitis, atopic dermatitis, and urticaria, were associated with adverse events after vaccination against COVID-19 [37]. Often, skin reactions after COVID-19 vaccines are heterogeneous. Most of them are mild to moderate and go away on their own [38]. Local skin reactions in atopic dermatitis patients in a Japanese population were less severe after the second dose of the COVID-19 vaccine [39].

While our study did not include vaccines such as Sinopharm or Sputnik V, studies from other regions have reported similar or different adverse event profiles. For instance, in China, inactivated vaccines showed a lower incidence of severe reactions compared to mRNA vaccines. A study conducted on the Chinese population showed that people suffering from allergic diseases are more susceptible to developing symptoms of COVID-19 infection and these symptoms were usually more severe [40]. Furthermore, in a study conducted in New York for patients with allergic disease, it was presented that allergic conditions were associated with an increased risk of receiving a COVID-19 diagnosis but reduced mortality after infection [41]. However, the meta-analysis showed that the risk of side effects after COVID-19 vaccination was higher in cases of atopic dermatitis patients, but the risk was reduced in psoriasis patients. Vaccinated patients with immune-related dermatological diseases had a lower risk of contracting COVID-19 than unvaccinated patients [42].

Special recommendations must be considered when prescribing vaccinations to the population taking biologics and Janus kinase (JAK) inhibitors utilized to treat atopic dermatitis, psoriasis, psoriatic arthritis, and alopecia areata [43].

Most patients with psoriasis reported no change in their psoriasis after vaccination, while 16.6% and 4.4% reported slight and severe worsening of the disease, respectively [44]. No adverse reactions were reported in the Italian population and no relapses of psoriasis were observed in the first weeks after vaccination [45]. This study showed the severity of psoriasis, confirmed based on patient’s observations in three people, and only in one person after medical consultation and the need to change pharmacotherapy. In the remaining nine people who suffered from psoriasis, contracting COVID-19 did not affect the course of psoriasis. In the Turkish population, no exacerbation of psoriasis was observed in patients who contracted COVID-19, but an exacerbation of psoriasis occurred after vaccination against COVID-19 in two patients [46].

In 70.3% of Chinese patients the progression of vitiligo after vaccination, mostly within 3 months was reported. In 55.6% of patients, disease progression was experienced after the second dose of vaccine. The results of the analysis showed that patients in the active phase had a higher risk of vitiligo and disease progression after COVID-19 infections and vaccinations [47]. Studies of other groups also showed an increasing number of cases reporting the appearance or severity of vitiligo after vaccination against COVID-19 [48]. In another study, 41.3% of patients with vitiligo had a progression of the disease within 1 week of vaccination, especially in older patients, in whom the time to onset of vitiligo was shorter [49]. The survey conducted among vitiligo patients did not show disease progression after contracting COVID-19 (6), and only one person experienced an exacerbation of vitiligo after vaccination.

The most common side effect after the first and second doses of the vaccine in the Polish population was pain at the injection site [50]. This is a known side effect of COVID-19 mRNA vaccines (such as Pfizer and Moderna vaccines) described as COVID Vaccine Arm (CVA). It appears within 5 to 10 days and is characterized by erythema and swelling at the vaccination site (usually around the deltoid muscle) and is sometimes associated with itching or pain. This reaction is harmless [51,52,53,54,55].

In a previously conducted study on the Polish population, it was noted that individuals who had previously contracted COVID-19 experienced worse AEFI after the first infection, while those who had not previously contracted COVID-19 experienced stronger AEFI after the second dose of the vaccine [13]. In the current study in the Polish population, there is a visible tendency to reduce the incidence of post-vaccination reactions with each subsequent dose of vaccine. A survey of hospital workers in Spain showed that local reactions occurred more often after the first dose of the vaccine, while systemic reactions occurred more often after the second dose [55]. In the Japanese population, both the incidence and severity of adverse events were higher after the second dose of vaccine than after the first dose. Comparing genders, women had a significantly higher incidence of side effects such as headache, skin pain, erythema, and itching. Younger age groups had a higher incidence of all adverse events except myalgia compared to older age groups [56]. Patients who experienced anaphylaxis were younger and more likely to have a history of allergy, asthma, and allergic rhinitis [57]. Short-term side effects of COVID-19 vaccines appear to be mainly local or transient, and old age and certain comorbidities (rheumatoid arthritis, intestinal, bone, and thyroid diseases) may increase susceptibility to side effects [58].

### Limitations

The study has several strengths and limitations worth highlighting. Among its strengths, the research employed a comprehensive online survey distributed across multiple universities, which enabled the collection of a substantial and diverse dataset with 513 complete responses. The focus on a specific population of students and university employees ensured a degree of homogeneity, which helped minimize potential confounding variables. Additionally, the study’s in-depth examination of the impact of COVID-19 and its vaccines on specific conditions such as atopic dermatitis and psoriasis adds valuable insights to a relatively underexplored area. Furthermore, the study’s detailed data collection, including demographic information and health outcomes related to COVID-19 infection and vaccination, allowed for a nuanced analysis of the issues at hand.

However, the study also has some limitations. The limited generalizability of the findings, primarily because the study focused on a specific population in Wrocław, poses a challenge, especially when considering different demographics or regions. Moreover, while the study attempted to isolate the effects of COVID-19 and its vaccination on skin diseases and comorbidities, there remains the potential for confounding factors such as pre-existing health conditions, lifestyle choices, and environmental influences that may not have been fully accounted for. Additionally, the limitations of this study include the focus primarily on vaccine reactions related to the Pfizer vaccine. This study highlights the ongoing issue of underreporting side effects after vaccination, raising concerns about inadequate monitoring by pharmaceutical companies. Moreover, the study underscores the effectiveness of the vaccine in preventing COVID-19 and demonstrates that the vaccine does not negatively impact comorbidities in the general population.

## 5. Conclusions

The study confirmed the safety of COVID-19 vaccines in individuals with comorbidities and skin diseases, such as vitiligo, psoriasis, and atopic dermatitis. Across all vaccine types, adverse events were most frequently reported after the first dose and decreased with subsequent doses. mRNA vaccines (Moderna and Pfizer) were associated with more significant second- and third-dose AEFIs, possibly due to the newer technology and slower adaptation of the body compared to the viral vector-based AstraZeneca vaccine. The data suggest that patients with comorbidities or chronic skin conditions can be safely vaccinated without exacerbating their underlying conditions. Despite the clear effectiveness of the vaccines, particularly in reducing COVID-19 severity, post-vaccination reactions remain underreported, highlighting the need for improved safety monitoring. Future research should focus on the long-term impact of vaccines on specific patient populations and expand the analysis to include less-represented vaccines, such as Johnson & Johnson.

## Figures and Tables

**Table 1:** Skin diseases occurring among respondents.

Skin Disease	Number of Participants	Participants Who Contracted COVID-19
Atopic dermatitis	47	32
Psoriasis	16	13
Acne vulgaris	54	38
Rosacea	16	6
Vitiligo	8	6
Alopecia areata	2	1

**Table 2:** Comorbidities occurring among respondents.

Comorbidities	Number of Participants	Participants Contracted COVID-19	Worsening of the Disease after Contracting COVID-19
Hashimoto’s disease	49	29	3
Heart disease/hypertension	50	29	5
Rheumatoid arthritis	7	3	2
Diabetes	12	3	1
Cancer	6	3	0
Atherosclerosis	6	4	0
Multiple sclerosis	1	0	0
Digestive disorders	2	2	1

**Table 3:** Skin diseases occurring in respondents, number of vaccinated, and disease worsening.

Skin Disease	Number of Participants	Vaccinated against COVID-19	Worsening of the Disease after Vaccination
Atopic dermatitis	47	41	8
Psoriasis	16	13	3
Acne vulgaris	54	47	5
Rosacea	16	15	0
Vitiligo	8	6	1
Alopecia areata	2	2	0

**Table 4:** Cases in which exacerbation of a comorbidity occurred after vaccination.

Comorbidities	Number of Participants	Vaccinated against COVID-19	Worsening of the Disease after Vaccination
Hashimoto’s disease	49	45	4
Heart disease/hypertension	50	48	7
Rheumatoid arthritis	7	7	2
Diabetes	12	12	1
Cancer	6	5	1
Atherosclerosis	6	5	0
Multiple sclerosis	1	1	0
Digestive disorders	2	2	1

## Data Availability

Aggregate data generated and/or during the current study are available in aggregate form in an Excel file and are available from the corresponding author upon reasonable request.

## References

[1] Awan M.H., Samreen S., Salim B., Gul H., Perveen S., Nasim A. (2022). Corona Virus Disease-19 Vaccine-Associated Autoimmune Disorders. Rheumatol. Immunol. Res..

[2] Fadlyana E., Rusmil K., Tarigan R., Rahmadi A.R., Prodjosoewojo S., Sofiatin Y., Khrisna C.V., Sari R.M., Setyaningsih L., Surachman F. (2021). A Phase III, Observer-Blind, Randomized, Placebo-Controlled Study of the Efficacy, Safety, and Immunogenicity of SARS-CoV-2 Inactivated Vaccine in Healthy Adults Aged 18–59 Years: An Interim Analysis in Indonesia. Vaccine.

[3] Fang X., Qiao S., Zhang R., Yang T., Wang Z., Kong Q., Sun M., Geng J., Fang C., Chen Y. (2023). Effects of Coronavirus Disease 2019 Vaccination on Seizures in Patients with Epilepsy. Chin. Med. J..

[4] Uwamino Y., Kurafuji T., Sato Y., Tomita Y., Shibata A., Tanabe A., Yatabe Y., Noguchi M., Arai T., Ohno A. (2022). Young Age, Female Sex, and Presence of Systemic Adverse Reactions Are Associated with High Post-Vaccination Antibody Titer after Two Doses of BNT162b2 MRNA SARS-CoV-2 Vaccination: An Observational Study of 646 Japanese Healthcare Workers and University Staff. Vaccine.

[5] Niebel D., Wenzel J., Wilsmann-Theis D., Ziob J., Wilhelmi J., Braegelmann C. (2021). Single-Center Clinico-Pathological Case Study of 19 Patients with Cutaneous Adverse Reactions Following COVID-19 Vaccines. Dermatopathology.

[6] El-Shitany N.A., Bagher A.M., Binmahfouz L.S., Eid B.G., Almukadi H., Badr-Eldin S.M., El-Hamamsy M., Mohammedsaleh Z.M., Saleh F.M., Almuhayawi M.S. (2022). The Adverse Reactions of Pfizer BioNTech COVID-19 Vaccine Booster Dose Are Mild and Similar to the Second Dose Responses: A Retrospective Cross-Sectional Study. Int. J. Gen. Med..

[7] Cantisani C., Chello C., Grieco T., Ambrosio L., Kiss N., Tammaro A., Tosti G., Paolino G., Pellacani G. (2022). Cutaneous Reactions to COVID-19 Vaccines in a Monocentric Study: A Case Series. J. Clin. Med..

[8] Sampath V., Rabinowitz G., Shah M., Jain S., Diamant Z., Jesenak M., Rabin R., Vieths S., Agache I., Akdis M. (2021). Vaccines and Allergic Reactions: The Past, the Current COVID-19 Pandemic, and Future Perspectives. Allergy Eur. J. Allergy Clin. Immunol..

[9] Rogers A., Rooke E., Morant S., Guthrie G., Doney A., Duncan A., MacKenzie I., Barr R., Pigazzani F., Zutis K. (2022). Adverse Events and Overall Health and Well-Being after COVID-19 Vaccination: Interim Results from the VAC4COVID Cohort Safety Study. BMJ Open.

[10] Cai C., Peng Y., Shen E., Huang Q., Chen Y., Liu P., Guo C., Feng Z., Gao L., Zhang X. (2021). A Comprehensive Analysis of the Efficacy and Safety of COVID-19 Vaccines. Mol. Ther..

[11] Kim M.A., Lee Y.W., Kim S.R., Kim J.H., Min T.K., Park H.S., Shin M., Ye Y.M., Lee S., Lee J. (2021). COVID-19 Vaccine-Associated Anaphylaxis and Allergic Reactions: Consensus Statements of the KAAACI Urticaria/Angioedema/Anaphylaxis Working Group. Allergy Asthma Immunol. Res..

[12] Romantowski J., Kruszewski J., Solarski O., Bant A., Chciałowski A., Pietrzyk I., Sańpruch P., Górska A., Chełmińska M., Knurowska A. (2022). Protocol of Safe Vaccination against COVID-19 in Patients with High Risk of Allergic Reactions. Clin. Transl. Allergy.

[13] Jęśkowiak I., Wiatrak B., Grosman-Dziewiszek P., Szeląg A. (2021). The Incidence and Severity of Post-Vaccination Reactions after Vaccination against COVID-19. Vaccines.

[14] Xu K., Wang Z., Qin M., Gao Y., Luo N., Xie W., Zou Y., Wang J., Ma X. (2023). A Systematic Review and Meta-Analysis of the Effectiveness and Safety of COVID-19 Vaccination in Older Adults. Front. Immunol..

[15] Du Y., Chen L., Shi Y. (2022). Safety, Immunogenicity, and Efficacy of COVID-19 Vaccines in Adolescents, Children, and Infants: A Systematic Review and Meta-Analysis. Front. Public Health.

[16] Wang Y., Zhang Y., Zhang M., Zhang X., Li H., Wang Y., Wang W., Ji J., Wu L., Zheng D. (2023). The Prevalence of Adverse Reactions among Individuals with Three-Dose COVID-19 Vaccination. J. Infect. Public Health.

[17] Zhang Y., Chen H., Lv J., Huang T., Zhang R., Zhang D., Luo L., Wei S., Liu X., Zhang S. (2022). Evaluation of Immunogenicity and Safety of Vero Cell-Derived Inactivated COVID-19 Vaccine in Older Patients with Hypertension and Diabetes Mellitus. Vaccines.

[18] Hoffmann M.A., Wieler H.J., Enders P., Buchholz H.G., Plachter B. (2021). Age- and Sex-Graded Data Evaluation of Vaccination Reactions after Initial Injection of the Bnt162b2 Mrna Vaccine in a Local Vaccination Center in Germany. Vaccines.

[19] Granados Villalpando J.M., de Romero Tapia S.J., del Baeza Flores G.C., Ble Castillo J.L., Juarez Rojop I.E., Lopez Junco F.I., Olvera Hernández V., Quiroz Gomez S., Ruiz Quiñones J.A., Guzmán Priego C.G. (2022). Prevalence and Risk Factors of Adverse Effects and Allergic Reactions after COVID-19 Vaccines in a Mexican Population: An Analytical Cross-Sectional Study. Vaccines.

[20] Gianfredi V., Minerva M., Casu G., Capraro M., Chiecca G., Gaetti G., Mazzocchi R.M., Musarò P., Basteri P., Bertini B. (2021). Immediate Adverse Events Following COVID-19 Immunization. A Cross-Sectional Study of 314,664 Italian Subjects. Acta Biomed..

[21] Duijster J.W., Lieber T., Pacelli S., Van Balveren L., Ruijs L.S., Raethke M., Kant A., Van Hunsel F. (2023). Sex-Disaggregated Outcomes of Adverse Events after COVID-19 Vaccination: A Dutch Cohort Study and Review of the Literature. Front. Immunol..

[22] Sánchez-Saez F., Peiró S., Cuenca L., Vanaclocha H., Limón R., Salas D., Burgos J.S., Sánchez-Payá J., Meneu R., Díez J. (2022). Side Effects during the Week after First Dose Vaccination with Four COVID-19 Vaccines. Results of the ProVaVac Survey Study with 13,837 People in Spain. Vaccine.

[23] Loosen S.H., Bohlken J., Weber K., Konrad M., Luedde T., Roderburg C., Kostev K. (2022). Factors Associated with Non-Severe Adverse Reactions after Vaccination against SARS-CoV-2: A Cohort Study of 908,869 Outpatient Vaccinations in Germany. Vaccines.

[24] Watanabe M., Balena A., Tuccinardi D., Tozzi R., Risi R., Masi D., Caputi A., Rossetti R., Spoltore M.E., Filippi V. (2022). Central Obesity, Smoking Habit, and Hypertension Are Associated with Lower Antibody Titres in Response to COVID-19 MRNA Vaccine. Diabetes Metab. Res. Rev..

[25] Chenchula S., Vidyasagar K., Pathan S., Sharma S., Chavan M.R., Bhagavathula A.S., Padmavathi R., Manjula M., Chhabra M., Gupta R. (2023). Global Prevalence and Effect of Comorbidities and Smoking Status on Severity and Mortality of COVID-19 in Association with Age and Gender: A Systematic Review, Meta-Analysis and Meta-Regression. Sci. Rep..

[26] Bots S.H., Riera-Arnau J., Belitser S.V., Messina D., Aragón M., Alsina E., Douglas I.J., Durán C.E., García-Poza P., Gini R. (2022). Myocarditis and Pericarditis Associated with SARS-CoV-2 Vaccines: A Population-Based Descriptive Cohort and a Nested Self-Controlled Risk Interval Study Using Electronic Health Care Data from Four European Countries. Front. Pharmacol..

[27] Couderc A.L., Ninove L., Nouguerède E., Rey D., Rebroin M., Daumas A., Tomasini P., Greillier L., Salas S., Duffaud F. (2022). Acceptance, Efficacy, and Safety of COVID-19 Vaccination in Older Patients with Cancer. J. Geriatr. Oncol..

[28] Shulman R.M., Weinberg D.S., Ross E.A., Ruth K., Rall G.F., Olszanski A.J., Helstrom J., Hall M.J., Judd J., Chen D.Y.T. (2022). Adverse Events Reported by Patients With Cancer After Administration of a 2-Dose MRNA COVID-19 Vaccine. JNCCN J. Natl. Compr. Cancer Netw..

[29] Weaver K.N., Zhang X., Dai X., Watkins R., Adler J., Dubinsky M.C., Kastl A., Bousvaros A., Strople J.A., Cross R.K. (2022). Impact of SARS-CoV-2 Vaccination on Inflammatory Bowel Disease Activity and Development of Vaccine-Related Adverse Events: Results From PREVENT-COVID. Inflamm. Bowel Dis..

[30] Miyazaki H., Watanabe D., Ito Y., Ikeda S., Okamoto N., Tokunaga E., Ku Y., Ooi M., Hoshi N., Kodama Y. (2023). Differences in Coronavirus Disease—19 Vaccination Related Side Effects in Patients with Ulcerative Colitis and Crohn’s Disease in Japan. Indian J. Gastroenterol..

[31] Wasserbauer M., Hlava S., Trojanek M., Stovicek J., Milota T., Drabek J., Koptová P., Cupkova A., Pichlerová D., Kucerova B. (2022). Efficacy and Safety of SARS-CoV-2 Vaccination in Patients with Inflammatory Bowel Disease on Immunosuppressive and Biological Therapy: Prospective Observational Study. PLoS ONE.

[32] An Z., Figueroa-Parra G., Zhou X., Li Y., Jaquith J., McCarthy-Fruin K., Sletten J., Warrington K.J., Weyand C., Crowson C.S. (2023). Immune Responses and Disease Biomarker Long-Term Changes Following COVID-19 MRNA Vaccination in a Cohort of Rheumatic Disease Patients. Front. Immunol..

[33] Li Y.K., Lui M.P.K., Yam L.L., Cheng C.S., Tsang T.H.T., Kwok W.S., Chung H.Y. (2022). COVID-19 Vaccination in Patients with Rheumatic Diseases: Vaccination Rates, Patient Perspectives, and Side Effects. Immun. Inflamm. Dis..

[34] Zeng H., Liu H., Liu Z., Zhou X., Lu X., Yan Z., Zhou Y., Dai L., Chen Y., Yang T. (2022). Safety and Immunogenicity of Inactivated COVID-19 Vaccination in Adult Rheumatic Patients in South China: A Prospective Study. Hum. Vaccin Immunother..

[35] Chen C., Huang S., Geng L., Lai P., Dou H., Zhang H., Chen H., Liang J., Sun L. (2024). COVID-19 Vaccination and Infection Status: A Cross-Sectional Survey of Patients with Rheumatic Diseases in China. Rheumatol. Int..

[36] Kridin K., Schonmann Y., Onn E., Bitan D.T., Weinstein O., Cohen A.D. (2022). Determinants and Effectiveness of BNT162b2 MRNA Vaccination Among Patients with Atopic Dermatitis: A Population-Based Study. Am. J. Clin. Dermatol..

[37] Yoon D., Jeon H.L., Noh Y., Choe Y.J., Choe S.A., Jung J., Shin J.Y. (2023). A Nationwide Survey of MRNA COVID-19 Vaccinee’s Experiences on Adverse Events and Its Associated Factors. J. Korean Med. Sci..

[38] Català A., Muñoz-Santos C., Galván-Casas C., Roncero Riesco M., Revilla Nebreda D., Solá-Truyols A., Giavedoni P., Llamas-Velasco M., González-Cruz C., Cubiró X. (2022). Cutaneous Reactions after SARS-CoV-2 Vaccination: A Cross-Sectional Spanish Nationwide Study of 405 Cases*. Br. J. Dermatol..

[39] Morimoto H., Hayano S., Ozawa N., Ogura Y., Usui H., Usami T., Ohse A., Otsuka M., Miyachi M., Tokura Y. (2021). Questionnaire Survey of Possible Association of Allergic Diseases with Adverse Reactions to Sars-Cov-2 Vaccination. Vaccines.

[40] Zhang H., Lin J., Wu J., Zhang J., Zhang L., Yuan S., Chen J., Tang Q., Zhang A., Cui Y. (2023). Allergic Diseases Aggravate the Symptoms of SARS-CoV-2 Infection in China. Front. Immunol..

[41] Chen C., Song X., Murdock D.J., Marcus A., Hussein M., Jalbert J.J., Geba G.P. (2024). Association between Allergic Conditions and COVID-19 Susceptibility and Outcomes. Ann. Allergy Asthma Immunol..

[42] Chirasuthat S., Ratanapokasatit Y., Thadanipon K., Chanprapaph K. (2024). Immunogenicity, Effectiveness, and Safety of COVID-19 Vaccines among Patients with Immune-Mediated Dermatological Diseases: A Systematic Review and Meta-Analysis. Acta Derm. Venereol..

[43] Hren M.G., Khattri S. (2024). Low Rates of Vaccination among Atopic Dermatitis, Alopecia Areata, Psoriasis, and Psoriatic Arthritis Patients on Biologics. Arch. Dermatol. Res..

[44] Wang Q., Lv C., Han X., Shen M., Kuang Y. (2021). A Web-Based Survey on Factors for Unvaccination and Adverse Reactions of SARS-CoV-2 Vaccines in Chinese Patients with Psoriasis. J. Inflamm. Res..

[45] Genovese G., Puig L., Di Lernia V., Graceea D., Bella D.O. (2022). Immunogenicity of Three Doses of Anti-SARS-CoV-2 BNT162b2 Vaccine in Psoriasis Patients Treated with Biologics. Front. Med..

[46] Kaya O., Keskinkaya Z., Mermutlu S.I., Oguz-Kilic S., Cakir H. (2023). COVID-19 Among Patients with Psoriasis: A Single-Center Retrospective Cross-Sectional Study. Infect. Dis. Clin. Microbiol..

[47] Shi X., Sun Y., Ding X. (2023). Impact of COVID-19 Vaccine and COVID-19 Infection on Vitiligo Activity and Progression. Clin. Cosmet. Investig. Dermatol..

[48] Tsai T.F., Ng C.Y. (2023). COVID-19 Vaccine–Associated Vitiligo: A Cross-Sectional Study in a Tertiary Referral Center and Systematic Review. J. Dermatol..

[49] Hou X., Wu N., Xu M., Kharel P., Wu F., Wu Y., Wang R., Chen J. (2023). Demographic and Clinical Feature Disparity between Progress and Non-Progress Patients with Vitiligo after COVID-19 Vaccination: A Cross-Sectional Study. Exp. Dermatol..

[50] Siewert B., Szabat A., Chądzińska-Cebula M., Purpurowicz-Miękus N., Sujkowski P., Spachacz R., Dworacki G., Wysocki J., Januszkiewicz-Lewandowska D., Gowin E. (2022). To vaccinate or not to vaccinate—bnt162b2 seroconversion rate and side effects among polish healthcare workers. Int. J. Occup. Med. Environ. Health.

[51] Fasano G., Bennardo L., Ruffolo S., Passante M., Ambrosio A.G., Napolitano M., Provenzano E., Nisticò S.P., Patruno C. (2022). Erythema Migrans-like COVID Vaccine Arm: A Literature Review. J. Clin. Med..

[52] Picone V., Martora F., Fabbrocini G., Marano L. (2022). “Covid Arm”: Abnormal Side Effect after Moderna COVID-19 Vaccine. Dermatol. Ther..

[53] Lindgren A.L., Austin A.H., Welsh K.M. (2021). COVID Arm: Delayed Hypersensitivity Reactions to SARS-CoV-2 Vaccines Misdiagnosed as Cellulitis. J. Prim. Care Community Health.

[54] Ramos C.L., Kelso J.M. (2021). “COVID Arm”: Very Delayed Large Injection Site Reactions to MRNA COVID-19 Vaccines. J. Allergy Clin. Immunol. Pract..

[55] Valera-Rubio M., Sierra-Torres M.I., Castillejo García R., Cordero-Ramos J., López-Márquez M.R., Cruz-Salgado Ó., Calleja-Hernández M.Á. (2022). Adverse Events Reported after Administration of BNT162b2 and MRNA-1273 COVID-19 Vaccines among Hospital Workers: A Cross-Sectional Survey-Based Study in a Spanish Hospital. Expert Rev. Vaccines.

[56] Maruyama A., Sawa T., Teramukai S., Katoh N. (2022). Adverse Reactions to the First and Second Doses of Pfizer-BioNTech COVID-19 Vaccine among Healthcare Workers. J. Infect. Chemother..

[57] Bian S., Li L., Wang Z., Cui L., Xu Y., Guan K., Zhao B. (2022). Allergic Reactions After the Administration of COVID-19 Vaccines. Front. Public Health.

[58] Nassar R.I., Alnatour D., Thiab S., Nassar A., El-Hajji F., Basheti I.A. (2022). Short-Term Side Effects of COVID-19 Vaccines: A Cross-Sectional Study in Jordan. Hum. Vaccin Immunother..

